# Drought Sensitivity of Spring Wheat Cultivars Shapes Rhizosphere Microbial Community Patterns in Response to Drought

**DOI:** 10.3390/plants12203650

**Published:** 2023-10-23

**Authors:** Jing Fang, Gongfu Shi, Shuli Wei, Jie Ma, Xiangqian Zhang, Jianguo Wang, Liyu Chen, Ying Liu, Xiaoqing Zhao, Zhanyuan Lu

**Affiliations:** 1 School of Life Science, Inner Mongolia University, Hohhot 010020, China; fangjing721@126.com (J.F.); y214575@126.com (G.S.); wslweishuli@163.com (S.W.); majie19952021@163.com (J.M.); liuying836354822@126.com (Y.L.); 2 Inner Mongolia Academy of Agricultural and Animal Husbandry Sciences, Hohhot 010031, China; zhangxiangqian_2008@126.com (X.Z.); wjg55998@163.com (J.W.); chenliyu1102@163.com (L.C.); 3 Key Laboratory of Black Soil Protection and Utilization (Hohhot), Ministry of Agriculture and Rural Affairs, Hohhot 010031, China; 4 Inner Mongolia Key Laboratory of Degradation Farmland Ecological Restoration and Pollution Control, Hohhot 010031, China

**Keywords:** drought stress, spring wheat, physiological parameters, soil characteristics, rhizosphere microbial diversity

## Abstract

Drought is the most important natural disaster affecting crop growth and development. Crop rhizosphere microorganisms can affect crop growth and development, enhance the effective utilization of nutrients, and resist adversity and hazards. In this paper, six spring wheat varieties were used as research material in the dry farming area of the western foot of the Greater Khingan Mountains, and two kinds of water control treatments were carried out: dry shed rain prevention (DT) and regulated water replenishment (CK). Phenotypic traits, including physiological and biochemical indices, drought resistance gene expression, soil enzyme activity, soil nutrient content, and the responses of potential functional bacteria and fungi under drought stress, were systematically analyzed. The results showed that compared with the control (CK), the leaf wilting, drooping, and yellowing of six spring wheat varieties were enhanced under drought (DT) treatment. The plant height, fresh weight (FW), dry weight (DW), net photosynthetic rate (Pn) and stomatal conductance (Gs), soil total nitrogen (TN), microbial biomass carbon (MBC), microbial biomass nitrogen (MBN), microbial biomass phosphorus (MBP), organic carbon (SOC), and soil alkaline phosphatase (S-ALP) contents were significantly decreased, among which, FW, Gs and MBC decreased by more than 7.84%, 17.43% and 11.31%, respectively. By contrast, the soil total phosphorus (TP), total potassium (TK), and soil catalase (S-CAT) contents were significantly increased (*p* < 0.05). *TaWdreb2* and *TaBADHb* genes were highly expressed in T.D40, T.L36, and T.L33 and were expressed at low levels in T.N2, T.B12, and T.F5. Among them, the relative expression of the *TaWdreb2* gene in T.L36 was significantly increased by 2.683 times compared with CK. Soil TN and TP are the most sensitive to drought stress and can be used as the characteristic values of drought stress. Based on this, a drought-tolerant variety (T.L36) and a drought-sensitive variety (T.B12) were selected to further analyze the changes in rhizosphere microorganisms. Drought treatment and cultivar differences significantly affected the composition of the rhizosphere microbial community. Drought caused a decrease in the complexity of the rhizosphere microbial network, and the structure of bacteria was more complex than that of fungi. The Shannon index and network modular number of bacteria in these varieties (T.L36) increased, with rich small-world network properties. Actinobacteria, Chloroflexi, Firmicutes, Basidiomycota, and Ascomycota were the dominant bacteria under drought treatment. The beneficial bacteria *Bacillus*, *Penicillium,* and *Blastococcus* were enriched in the rhizosphere of T.L36. *Brevibacillus* and *Glycomyce* were enriched in the rhizosphere of T.B12. In general, drought can inhibit the growth and development of spring wheat, and spring wheat can resist drought hazards by regulating the expression of drought-related genes, regulating physiological metabolites, and enriching beneficial microorganisms.

## 1. Introduction

In the farmland ecosystem, crops, soil, and microbes interact with each other to maintain the balance of the farmland ecosystem [1,2]. Rhizosphere microbes play an important role in the decomposition of soil organic matter, efficient nutrient utilization, and material recycling and can also improve crop tolerance in adversity and stress, inhibit the occurrence of pathogens, and regulate host immunity [3,4,5,6]. Therefore, this complex plant-associated microbial community is also known as the second genome of the plant [2,5]. Studies have reported that similar environments have similar dominant microbial preferences, and corresponding genes in the genome of dominant microorganisms can improve their adaptability to the environment. For instance, the presence of 18 genes in the genome of dry land microorganisms can improve their tolerance to drought or salinity stress [7,8,9,10]. Plants can secrete organic acid, flavonoids, amino acids, and other root products and selectively “domesticate” specifically beneficial microorganisms to their rhizosphere [11,12,13], which can affect the composition and diversity of the rhizosphere microbial community [14,15]. Therefore, understanding the influence of environmental stress on the microbial community of crop soil from the aspects of the crop, soil microenvironment, and microbial organisms is conducive to improving crop stress resistance and sustainable agricultural development.

Drought, as the most severe natural disaster affecting the growth and development of crops, has become one of the major factors limiting crop production globally. Overall, crop yield reduction exceeds the combined yield reduction caused by other factors [16,17]. When plants are under drought stress, their antioxidant system and osmotic regulation system make positive responses to resist drought hazards [18,19]. For example, the key antioxidant enzymes (POD, SOD) of the antioxidant system and the key water-soluble substances (Pro) of the osmotic regulation system have an increasing trend, which can prevent the accumulation of reactive oxygen species (ROS) and reduce the water loss of leaf tissue, thereby improving the drought resistance of crops [18,20]. The availability of soil nutrients is also a key factor in determining plant growth. Drought stresses not only limit the migration of soil nutrients but also lead to a significant reduction in soil microbial content [21]. Studies have shown that drought stress can significantly reduce the photosynthetic rate of plants, change the accumulation and transformation process of organic matter in plants, and thus reduce the efficiency with which plants utilize N and P elements in the soil [22,23].

Rhizosphere microbes play an important role in farmland ecosystems, which can actively influence crops by participating in biogeochemical cycling through the process of organic matter decomposition and mineralization and increasing the bioavailability of soil nutrients [24,25]. Ochoa-Hueso et al. [26] revealed that drought can significantly change the structure of bacterial and fungal communities in soil and promote the enrichment of drought-tolerant microorganisms. Plant growth-promoting rhizobacteria (PGPR) can indirectly or directly promote the expression of gene tolerance in plant drought resistance [27,28]. The PGPR *Bacillus amyloliquefaciens*, *Bacillus licheniformis*, *Bacillus thuringiensis*, and *Bacillus subtilis* increase maize biomass, leaf relative water content, the soil/root tissue ratio of root attachment, and aggregate stability, thus enhancing maize tolerance to drought stress [19]. The interaction of *Klebsiella* sp., *Enterobacter ludwigii*, and *Flavobacterium* sp. can positively affect various growth parameters, water status, membrane integrity, osmolyte accumulation, and stress response gene expression and improve drought tolerance in wheat [29]. Under water stress, wheat uses *Azospirillum* sp. to reduce adverse hazards [30]. In conclusion, numerous studies have shown that drought-tolerant plant species are diverse in drought-resistant microbes in the rhizosphere. There are also differences within species (different genotypes) in the drought-resistant microbial community structure in the rhizosphere, such as disease resistance and high sensitivity, drought resistance, and water sensitivity. The abundance of microbial species was different in resistant and susceptible varieties of tomato [31]. Therefore, an analysis of microbial community structure changes in screening and identifying the species and functions of soil microorganisms are effective methods to study the mechanism of stress resistance in crops.

Drought tolerance in plants is a complex trait that is significantly influenced by the environment [32]. The correlation study of crop phenotype and microbial diversity and the use of microorganisms to improve resistance to crop stress and reduce adverse damage is an important direction of crop-stress-resistance research. Spring wheat is one of the main crops planted in northern China [33], and both drought-resistant spring wheat varieties and water-sensitive varieties have their own preferred specific microorganisms. Previous studies on the drought resistance of wheat have performed much work in physiological [34,35], molecular [27,36], and other aspects. However, few related studies have analyzed their drought resistance mechanisms from the perspective of microorganisms. We hypothesized that drought stress can affect the microbial community structure, reduce microbial network complexity, and enrich drought-resistant microbial groups. Therefore, in this study, two water control treatments, including rainproof dry sheds and the regulation and replenishment of water, were carried out, and the preliminary screening of six wheat varieties was performed on two drought-responsive and contrasting wheat varieties. High-throughput sequencing technology combined with bioinformatics was used to (1) explore the diversity and community structure of bacterial and fungal communities in the rhizosphere soil of spring wheat; (2) clarify dominant bacterial and fungal communities in the rhizosphere soil of different drought-resistant spring wheat varieties; and (3) reveal the response mechanism of rhizosphere microorganisms of spring wheat to drought stress. The aim was to provide data support and a theoretical basis for the research and development of highly resistant microbial fertilizer and the improvement of farmland soil in arid areas.

## 2. Materials and Methods

### 2.1. Experimental Materials and Field Experimental Design

Six different drought-resistant spring wheat varieties were used in this study: T.N2, T.F5, T.B12, T.L33, T.L36, and T.D40 (Table S1). The experiment was carried out in 2019 at the Tenihe Soil Management and Ecological Restoration Scientific Observation and Experimental Station of the Inner Mongolia Autonomous Region Academy of Agricultural and Animal Husbandry Sciences (Tenihe Farm: 49°55′ N, 120°48′ E, altitude 650 m, frost-free period 90~105 d, annual average sunshine hours 2589 h). The experimental area is located in the temperate semiarid continental grassland climate, and the soil type is chernozem, which is a typical black soil representative of the western foothills of Daxing’ angling in the agro-pastoral ecotone of Inner Mongolia. The basic nutrient content of 0–20 cm soil was as follows: available nitrogen 115 mg kg^−1^, available potassium 144 mg kg^−1^, available phosphorus 20 mg kg^−1^, and pH 6.8. The average annual precipitation of Tenihe from 2000 to 2019 was 334.86 mm (data collected from the China Meteorological Data Service Centre), and precipitation during the whole growth period of spring wheat in 2019 was 223.9 mm (Figure S1).

There were 12 treatments in the experiment. The main treatment included two types of water treatment: a drought treatment (DT, 0–20 cm soil mass water content 8~12%) and a control treatment (CK, 0–20 cm soil mass water content 25~30%); the sub treatments were 6 different spring wheat varieties (Table S1). The DT and CK were carried out in the dry shed, and the soil water content was strictly controlled by a dry shed rain control to regulate water replenishment in the dry shed. Mechanical sowing was carried out on 5 May 2019. The spring wheat sowing amount was 300 kg·hm^−2^, urea was 60 kg·hm^−2^, diammonium phosphate was 180 kg·hm^−2^, and potassium sulfate was 30 kg·hm^−2^ which were all applied as seed fertilizer, and no topdressing was applied in the later stage. Except for water treatment, the other management methods were the same as those in these fields. A randomized block design was used in the experiment. Each treatment was repeated three times for a total of 36 plots. Each plot area was 9 m^2^, the block spacing was 0.5 m, and a 1 m wide protection row was set. The drought treatment lasted for a total of 22 days, from the jointing stage of spring wheat (4 July) to the end of the flowering stage of spring wheat (25 July). In the 22 days of drought treatment, soil mass water content monitoring was carried out 5 times. The final soil mass water content of the drought treatment was 10.83%, and that of the control was 29.12%. The specific monitoring time is shown in Figure S2.

### 2.2. Determination of Soil Mass Water Content

The ‘S’-type sampling method was used to sample the soil mass water content. The soil samples of 0–20 cm for the soil layer were auger boring with soil, repeated three times, and the soil mass water content was determined using the drying method. The aluminum box with soil samples was placed in a 105 °C oven drying oven at a constant weight, and according to the following formula, calculated the soil mass water content [37].

Calculation formula: w (%) = (g2 − g3)/(g3 − g1) × 100

In this formula, w is the soil mass water content (%); g1 is the mass of the aluminum box (g); g2 is the mass of the aluminum box + wet soil (g); g3 is the mass of the aluminum box + dried soil sample (g).

### 2.3. Sample Collection and Preservation

The experiment was conducted on July 26 for sample collection. Plant samples: nine representative spring wheat plants were randomly selected in each plot, and each treatment was repeated three times. The plants were placed on ice and transported to the laboratory. The flag leaves of spring wheat were cut off with sterile scissors, washed with sterile water, dried with absorbent paper, and placed in a 10 mL sterile tube. Then, fresh young and vigorous roots of spring wheat were selected, and the attachments on the surface of the roots were quickly washed with sterile water. After ultrasonic washing several times, the residual sterile water on the surface of the roots was dried with absorbent paper, and the young roots were cut into 1 cm pieces with sterile scissors. The three repeated spring wheat root samples were mixed with the same amount and placed in a 5 mL sterile centrifuge tube. The above samples were labeled, frozen in liquid nitrogen, and stored in a refrigerator at −80 °C. The flag leaf samples of spring wheat were used to determine plant physiological indices, and the root samples were used to determine drought resistance gene expression.

Soil samples: a 5-point sampling method was used in each plot to remove floating soil on the surface of the soil, and soil samples of the 0–20 cm soil layer were collected using a soil auger. The sampling of each treatment was repeated three times. After the soil samples were fully and evenly mixed, they were divided into two parts. A portion of the soil sample was sieved with a 1 mm sterile mesh sieve, dried in the shade, and stored in a sealed plastic bag containing desiccant to determine soil enzyme activity. Another part of the soil samples was placed into plastic bags and immediately stored in a refrigerator at 4 °C for soil microbial biomass determination. Five representative spring wheat plants were randomly selected in each plot. The bulk soil of the root was removed, and approximately 1 mm of soil was retained on the root and placed on ice for transportation to the laboratory. The wheat roots were placed in a sterile tube, washed with a PBS buffer, and centrifuged to obtain rhizosphere soil. Rhizosphere soil samples were labeled, frozen in liquid nitrogen, and stored in a refrigerator at −80 °C. Rhizosphere soil samples were collected for subsequent microbial diversity sequencing.

### 2.4. Determination of Physiological and Biochemical Characteristics of Plants

The physiological and biochemical characteristics of spring wheat plants were mainly measured using whole plant fresh weight (FW), whole plant dry weight (DW), net photosynthetic rate (Pn), stomatal conductance (Gs), peroxidase (POD), proline content (Pro) and other indicators. After the soil mass water content of the drought treatment reached the expected level, 5 representative spring wheat plants with the same vigor of growth were selected in each plot, and the FW was determined. After the fresh weight of spring wheat was weighed, it was placed in the oven at 105 °C for 0.5 h and then dried at 80 °C until constant weight and its DW was determined. The red and blue light sources were set to 1200 μmol·m^−2^·s^−1^ from 9:00 to 11:30 in the morning using a portable photosynthesis system (LI-6800, LI-COR, Lincoln, NE, USA). The Pn and Gs of three spring wheat flag leaves with uniform growth in each plot were measured [38]. POD was determined using the guaiacol method [39], and Pro was determined via the sulfosalicylic acid extraction method [40].

### 2.5. Analysis of Drought Resistance Gene Expression in the Root of Spring Wheat

The total RNA of different varieties of spring wheat roots under control and drought treatment was extracted using the Easy Pure Plant RNA Kit (catalog number ER301, Beijing TransGen Biotech Co., Ltd., Beijing, China). The concentration and quality of RNA were detected by agarose gel electrophoresis, a gel imaging system (Gel Doc XR+, Hercules, CA, USA), and an ultramicro nucleic acid analyzer (NanoDrop 2000, Waltham, MA, USA). According to the method of TransScript All-in-One First-Strand cDNA Syn One-Step gDNA Removal thesis SuperMix for qPCR (One-Step gDNA Removal) kit (catalog number AT341, Beijing TransGen Biotech Co., Ltd.), the total RNA was reverse-transcribed into cDNA and stored in an ultra-low temperature refrigerator at−80 °C. According to the wheat *TaWdreb2* (GenBank No. AB193608.1), *TaBADHb* (GenBank No. Sequence information of AY050316.1) gene and wheat watchman gene Actin (GenBank No. AK458303.1), an internal reference was used to design a primer for qRT-PCR using Primer5.0 software (Table S2). Using cDNA as a template, qRT-PCR was performed using the TransStart Tip Green qPCR SuperMix kit (catalog number AQ601, Beijing TransGen Biotech Co., Ltd.). The qRT-PCR reaction system (20 μL): cDNA (100 ng·μL^−1^) 1 μL, Forward Primer and Reverse Primer (10 μM) 0.8 μL, 2 × PerfectStartTMGreen qPCR SuperMix 10 μL, Nuclease-free Water 7.4 μL. The qRT-PCR amplification was performed using a fluorescence quantitative PCR instrument (Light Cycler 4800II, Basle, Switzerland) [41], and the reaction procedure was as follows: 95 °C 30 s; 95 °C 5 s, 60 °C 30 s, 40 cycles, where 3 biological replicates and 3 technical replicates were set for each sample.

### 2.6. Determination of Soil Chemical Properties and Microbiological Properties

The soil’s chemical properties were mainly measured by organic carbon (SOC), total nitrogen (TN), total phosphorus (TP), and total potassium (TK) [42]. SOC was determined using the potassium dichromate oxidation-diluted heat method. TN content was determined using the semi-micro Kjeldahl method. The content of TP was determined using the HClO_4_-H_2_SO_4_ method. The TK content was determined using the NaOH melting-flame photometer method. Soil microbiological properties were determined by catalase (S-CAT), alkaline phosphatase (S-ALP) [43], microbial biomass carbon (MBC), microbial biomass nitrogen (MBN), and microbial biomass phosphorus (MBP) [44]. The S-CAT was determined via potassium permanganate titration. Alkaline phosphatase activity was determined using the disodium phenyl phosphate colorimetric method. MBC was determined via chloroform fumigation extraction-volume analysis. The content of MBN was determined using the chloroform fumigation extraction-ninhydrin colorimetric method. MBP was determined by chloroform fumigation extraction and total phosphorus determination. Three independent replicates were set up to determine each soil index in each sample.

### 2.7. Soil Total DNA Extraction and 16S and ITS rRNA Sequencing

According to the procedures of the DNeasy^®^PowerSoil^®^Pro Kit (Article No.47014, QIAGEN Germany, Dusseldorf, Germany), the total DNA of rhizosphere soil samples for different spring wheat varieties was extracted. The total DNA concentration and quality were detected by 1% agarose gel electrophoresis (100 V, 40 min) and an ultramicro nucleic acid analyzer (NanoDrop 2000, Waltham, MA, USA). The hypervariable region V4 of the bacterial 16S rRNA gene was amplified using 515F (5′-GTGCCAGCMGCCGCGGTAA-3′) and 806R (5′-GGACTACHVGGGTWTCTAAT-3′) primers [45]. Fungal ITS sequences were amplified using ITS1F (5′-CTTGGTCATTTAGAGGAAGTAA-3′) and ITS2R (5′-GCTGCGTTCTTCATCGATGC-3′) primers [46,47]. The total PCR amplification system was 30 μL: 15 μL for the Phusion Master Mix (2×), 3 μL for the Primer (2 μM), 10 μL for gDNA (1 ng·μL^−1^), and 2 μL for ddH_2_O. The PCR amplification reaction conditions were as follows: 1 × (initial denaturation 98 °C 1 min); 30 × (denaturing 98 °C 10 s; annealing 50 °C 30 s; extension 72 °C 30 s); 1 × (single extension 72 °C 5 min); finally, they were stored at 4 °C (PCR instrument: Bio-Rad T100, Hercules, USA). The PCR products were assessed using 2% agarose gel electrophoresis. The PCR product was purified by 1 × TAE 2% agarose gel electrophoresis, and the target band (400~450 bp) was recovered using the GeneJET gel recovery kit (Thermo Scientific, Waltham, MA, USA). The library was constructed using the Illumina TruSeq DNA PCR-Free Library Preparation Kit. After Qubit quantification and library testing, the constructed library was sequenced using NovaSeq 6000 (Novogene Co., Ltd., Beijing, China). Raw data were uploaded to the NCBI SRA database (accession number: PRJNA995401).

### 2.8. High-Throughput Sequencing Data Analysis and Statistical Analysis

Fastp software (https://github.com/OpenGene/fastp, version 0.19.6, accessed on 1 August 2019) was used to perform quality control on the double-ended original sequences [48]. FLASH software (http://www.cbcb.umd.edu/software/flash, accessed on 1 August 2019, version 1.2.11) was used for splicing, low-quality read sequences were filtered out, and the obtained double-ended sequence data were spliced into tags [49]. Using UPARSE software (http://drive5.com/uparse/, accessed on 1 August 2019, version 7.1), OTU (operational taxonomic unit) clustering and chimaera elimination were performed on the quality control spliced sequence based on 97% similarity [50,51]. The RDP classifier (http://rdp.cme.msu.edu/, accessed on 1 August 2019, version 2.11) and UNITE gene database (https://unite.ut.ee, accessed on 1 August 2019) were used to compare the 16S rRNA sequence and ITS sequence for OTU species taxonomic annotation, and the confidence threshold was 70% [52,53].

All data analysis was performed on the NoVoMagic Cloud platform (https://magic.novogene.com/customer/main #/homeNew, accessed on 1 October 2019) as follows: the alpha diversity Shannon index [54] was calculated using Qiime software (Version 1.7.0), and the Wilcoxon rank sum test was used to analyze the differences between groups for alpha diversity. Principal coordinate analysis (PCoA) based on the Bray‒Curtis distance algorithm was used to test the similarity of the microbial community structure between samples, and the PERMANOVA nonparametric test was used to analyze whether the difference in the microbial community structure between samples was significant. LEfSe (linear discriminant analysis effect size) analysis (http://huttenhower.sph.harvard.edu/LEfSe, accessed on 1 October 2019) (LDA > 3.9, *p* < 0.05) was used to determine bacterial groups with significant differences in abundance from the phylum to genus level in different drought-resistant spring wheat varieties [55]. According to the relative abundance information of OTUs, a test of spring wheat species abundance between the control and drought treatments was carried out. The x-axis is the average relative abundance of log10 transformation, and the y-axis is the abundance multiple for the two comparison groups. The figure shows *p* < 0.01 and a fold change at >1 or <−1 points with differences in abundance; we counted the number and then used ggplot2 software to map the volcano [56]. Based on Spearman correlation |r| > 0.8 *p* < 0.01, collinearity network analysis was performed on the top 100 OTUs [56,57]. The nodes in the network represented an OTU, and the edges represented the correlation between two OTUs. The ‘igraph’ and ‘psych’ packages in R were used to determine the relationship between OTUs and Gephi (https://gephi.org/, accessed on 1 May 2023), which was used for network visualization. Topological features of networks (such as modularity, average degree, and average clustering coefficient) are designed to describe the network structure [58]. Statistical analysis was performed using IBM SPSS Amos 22.0 (USA). One-way analysis of variance (ANOVA) and Duncan’s multiple comparison method were used to test the significance of differences in spring wheat plant physiology and soil physical and chemical indicators (*p* ≤ 0.05). GraphPad Prism 9 and Origin Pro2021 were used for visualization.

## 3. Results

### 3.1. Response of Physiological Parameters of Spring Wheat Varieties with Different Drought Resistance to Drought

**Figure 1:**
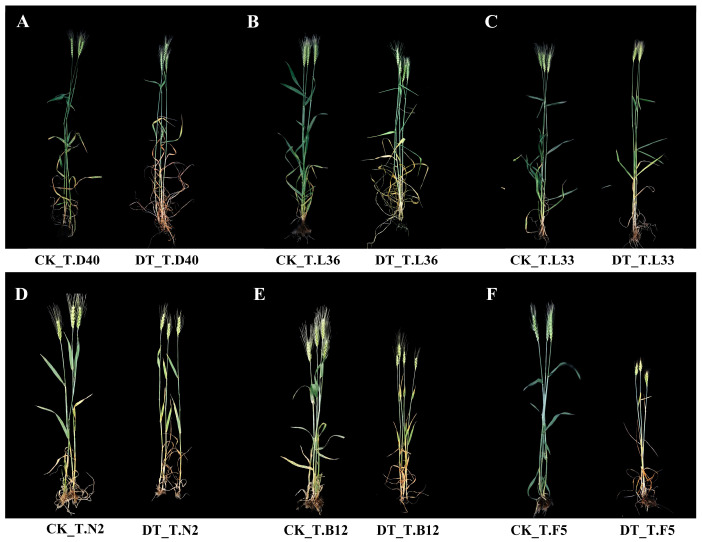
Morphological characteristics of different spring wheat varieties under drought stress. (**A**) Comparison of T.D40 plant height under control (CK) and drought (DT) treatment. (**B**) Comparison of T.L36 plant height under CK and DT treatment. (**C**) Comparison of T.L33 plant height under CK and DT treatment. (**D**) Comparison of T.N2 plant height under CK and DT treatment. (**E**) Comparison of T.B12 plant height under CK and DT treatment. (**F**) Comparison of T.F5 plant height under CK and DT treatment.

**Figure 2:**
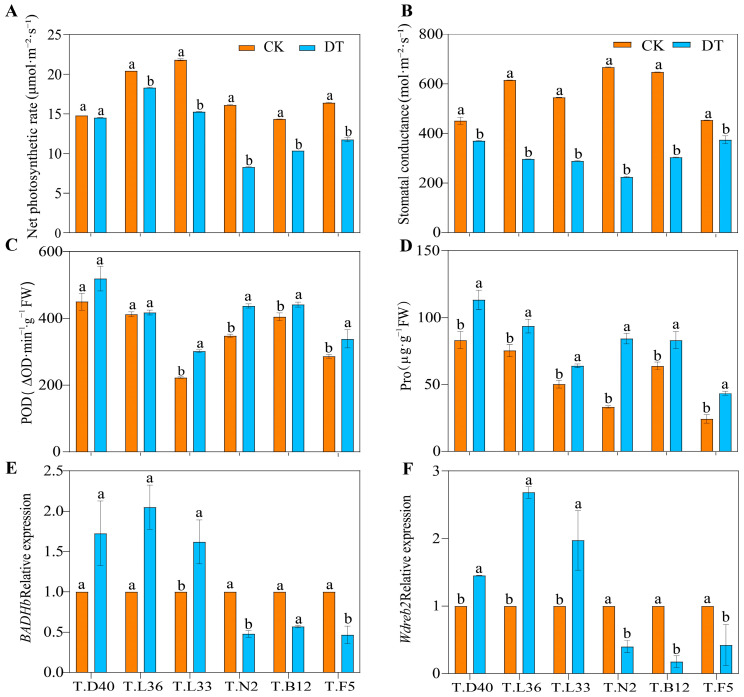
Changes in physiological parameters of different spring wheat varieties under drought stress. (**A**) Net photosynthetic rate (Pn). (**B**) Stomatal conductivity (Gs). (**C**) Peroxidase activity (POD). (**D**) Proline content (Pro). (**E**) *TaBADHb* Relative expression. (**F**) *TaWdreb2* Relative expression. Different letters indicate significant differences at the 0.05 level.

In our study, different varieties of spring wheat were treated with control (CK), and drought (DT) conditions, and the responses of plants to drought were systematically studied from the aspects of plant phenotypic changes, physiological characteristics, and gene expression. The results showed that compared with those under the control (CK), the leaf wilting, drooping, and yellowing of spring wheat plants was enhanced under drought (DT) treatment, and plant height was significantly decreased (Figure 1). Under DT treatment, the fresh weight (FW) and dry weight (DW) of spring wheat decreased significantly (*p* < 0.05), and FW decreased by more than 7.84%. In six spring wheat varieties, the decrease in FW and DW of T.L36 was the smallest, which was 7.87% and 15.48%, respectively (Figure S3A,B). The DT treatment significantly decreased the net photosynthetic rate (Pn) and stomatal conductance (Gs) of spring wheat flag leaves (*p* < 0.05), among which the Pn change amplitude of various varieties in order of magnitude was T.N2 > T.L33 > T.F5 > T.B12 > T.L36 > T.D40 (Figure 2A). The decrease in Gs was more than 17.43%, and the maximum decrease in Gs in T.N2 was 66.34% (Figure 2B). Compared with CK, the peroxidase (POD) activity and proline (Pro) content of spring wheat flag leaves under DT treatment increased significantly (*p* < 0.05), among which T.L36 varieties increased by 1.29% and 24.24%, respectively (Figure 2C,D). The results show that drought affected the normal growth and development of spring wheat, and the drought resistance of different spring wheat varieties was different. Spring wheat can maintain its normal physiological function by regulating the contents of Pn, Gs, POD, Pro, and other indicators.

Under drought stress, the phenotypic, physiological, and biochemical characteristics of crops change significantly, and these changes can be related to gene regulation. Egawa et al. [59] and Niazian et al. [60] found that *TaBADHb*, *TaWdreb2*, and other genes change significantly under drought stress in wheat. To further explore the changes in spring wheat under drought stress, we used RT‒qPCR technology to study *TaBADHb* and *TaWdreb2* genes at the molecular level. Compared with CK, the relative expression levels of the *TaBADHb* and *TaWdreb2* genes in the roots of T.D40, T.L36, and T.L33 spring wheat varieties increased significantly under the DT treatment (*p* < 0.05), while T.N2, T.B12, and T.F5 showed the opposite results (Figure 2E,F). The relative expression levels of *TaBADHb* in different spring wheat varieties were in the order of T.L36 > T.D40 > T.L33 > T.B12 > T.N2 > T.F5 (Figure 2F). The relative expression of the *TaWdreb2* gene in T.L36 was 2.683 times higher than that in CK, while the relative expression of *TaWdreb2* in T.F5 was 0.422 times lower than that in CK (Figure 2E). Therefore, the expression level of drought resistance genes can also reflect the fact that the drought resistance of spring wheat with an upregulated expression of drought resistance genes may be stronger than that of low-expression varieties.

### 3.2. Effects of Drought Stress on Soil Indices of Spring Wheat Varieties with Different Drought Resistance

**Figure 3:**
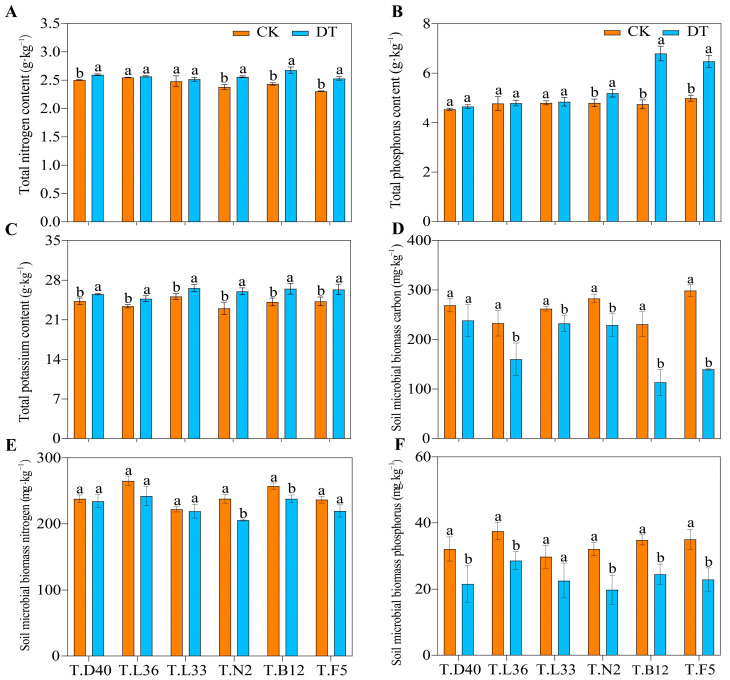
Effects of drought stress on soil indexes of spring wheat. (**A**) Total nitrogen content (TN). (**B**) Total phosphorus content (TP). (**C**) Total potassium content (TK). (**D**) Soil microbial biomass carbon (MBC). (**E**) Soil microbial biomass nitrogen (MBN). (**F**) Soil microbial biomass Phosphorus (MBP). Different letters indicate significant differences at the 0.05 level.

To further clarify the effects of drought stress on different spring wheat varieties, we also studied the soil nutrient content, microbial biomass, enzyme activity, and other indicators of spring wheat (Figure 3 and Figure S4). Compared with CK, DT treatment significantly reduced the soil’s total nitrogen (TN) (Figure 3A), soil microbial biomass carbon (MBC), soil microbial biomass nitrogen (MBN), soil microbial biomass phosphorus (MBP), soil organic carbon (SOC) content, and alkaline phosphatase (S-ALP) activity (Figure 3D,F and Figure S4A,C) in six spring wheat fields. However, the soil total phosphorus (TP), total potassium (TK), and soil catalase (S-CAT) contents increased significantly (*p* < 0.05) (Figure 3B,C and Figure S4B).

Among them, under drought stress, the T.B12 soil TN content decreased by 8.83%, while that of T.L36 decreased by 0.78% (Figure 3A). The maximum TP content of T.B12 soil under the DT treatment was 6.80 g·kg−1 (Figure 3B). T.N2 soil TK content increased by 13.02% (Figure 3C). The decreasing order of the MBC content in six spring wheat varieties was T.F5 > T.B12 > T.L36 > T.N2 > T.D40 > T.L33 (Figure 3D). The soil MBN content of T.L33 decreased by 1.28%, and the soil MBP content of T.L36 decreased by 23.77%. The soil SOC of T.B12 decreased by 15.41% (Figure S4A). The maximum increase in S-CAT in BM12 soil was 9.55% (Figure S4B), while its S-ALP decreased by 14.51% (Figure S4C). Under drought treatment, the soil indices of the spring wheat field changed significantly. Among the soil indices, the change range of T.B12 was larger, and the change ranges of LM36 and LM33 were smaller. Therefore, different spring wheat varieties have different levels of resistance to drought stress. The varieties with strong resistance can balance soil nutrients by adjusting the content of each soil index, which is conducive to their absorption and utilization of nutrients.

### 3.3. Screening and Selection of Spring Wheat Genotypes for Drought Tolerance and Sensitivity

**Figure 4:**
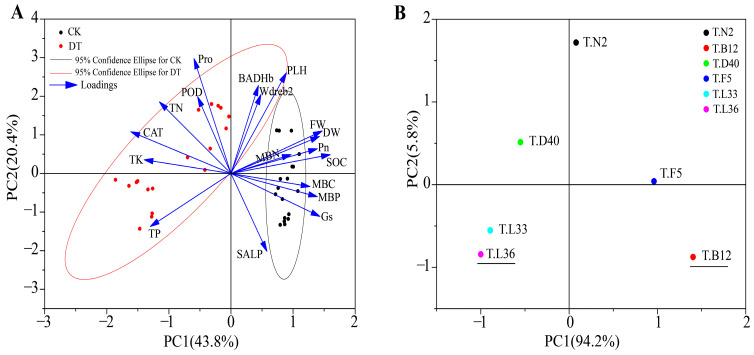
Principal component analysis of each index of 6 spring wheat varieties under drought treatment. (**A**) Principal component analysis of each index. (**B**) Categorization of different spring wheat genotypes for drought tolerance and sensitivity.

The drought had a significant effect on the changes in physiological parameters and soil indices of different spring wheat varieties. To further explore the degree of influence of drought on each index of different spring wheat varieties, principal component analysis (PCA) was used (Figure 4). The results show that the distribution of PCA on the PC1 and PC2 axes after DT treatment was 43.8% and 20.4%, respectively. The spring wheat samples treated with CK and DT were clearly distinguished, and it was further clarified that DT treatment was the main factor causing differences in various indicators of different spring wheat varieties. DT treatment had the greatest impact on TN and TP (Figure 4A).

We further analyzed the changes in TN and TP in different spring wheat varieties under drought treatment and carried out a graphical PCA on variety similarity and separation. PCA after DT treatment was distributed at 94.2% and 5.8% on the PC1 and PC2 axes, respectively (Figure 4B). Among the six varieties, T.L36 and T.B12 had the highest segregation. Therefore, based on the effects of drought stress on TN and TP, we selected two varieties, T.L36 and T.B12, to analyze the differences in rhizosphere microbial diversity and community changes between these two varieties under drought stress.

### 3.4. Alpha Diversity of Rhizosphere Soil Microorganisms in Spring Wheat

To clarify the differences in rhizosphere microbial communities between T.L36 drought-tolerant varieties and T.B12 drought-sensitive varieties under drought stress, we first divided the bacterial and fungal OTUs according to a similarity level greater than 97% and performed cluster analysis. The dilution curve of the sample tended to flatten with increasing sequencing data, indicating that the sequencing data were reasonable and could cover bacterial (Figure S5A) and fungal communities (Figure S5B). We also studied the alpha diversity of bacteria and fungi in rhizosphere soil samples of the T.L36 drought-tolerant variety and T.B12 drought-sensitive variety under drought stress and found that there was no significant difference in the Shannon index between different treatments of the same variety (*p* > 0.05) (Table 1). In the bacterial alpha diversity, compared with CK, the Shannon index of T.L36 and T.B12 showed an increasing trend under DT treatment, which increased by 23.76% and 22.86%, respectively, where the increase in T.L36 was larger. In the fungal alpha diversity, the Shannon index of T.L36 increased by 3.05% under DT treatment, while that of T.B12 decreased by 5.57%. Therefore, the bacterial diversity in the rhizosphere of spring wheat increased significantly under drought treatment, but the fungal diversity of drought-sensitive varieties decreased (Table 1).

### 3.5. Microbial Community Composition of Spring Wheat Rhizosphere Soil

We used the Bray‒Curtis algorithm in principal coordinate analysis (PCoA) and the PERMANOVA test to evaluate the factors affecting changes in bacterial and fungal communities in the rhizosphere soil of T.L36 drought-tolerant varieties and T.B12 drought-sensitive varieties, respectively (Figure S6). Compared with CK, the DT treatment demonstrated significant differences in the community structure of bacteria (*p* = 0.002) and fungi (*p* = 0.001) in the rhizosphere soil of T.L36 and T.B12 varieties, and the soil samples of T.L36 and T.B12 varieties under CK treatment were far from the DT treatment sample points (Figure S6A,B). Therefore, water conditions are the main factors affecting microbial community differences in the rhizosphere soil samples of spring wheat, and varieties are secondary factors. In addition, the PERMANOVA test confirmed that the effect of drought treatment on the bacterial (R^2^ = 0.599) community composition was greater than that on the composition of the fungal community (R^2^ = 0.494).

**Figure 5:**
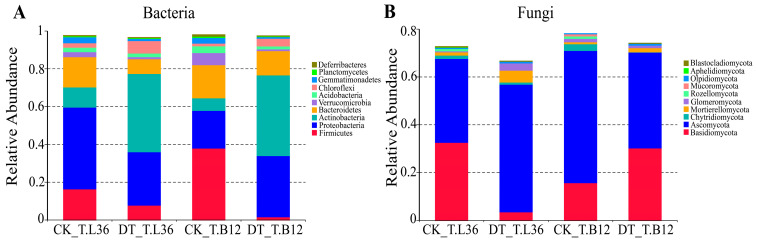
T.L36 and T.B12 under drought stress species composition at the phylum level. (**A**) Species composition of rhizosphere bacteria at the phylum level. (**B**) Species composition of rhizosphere fungi at the phylum level.

The species of rhizosphere bacterial and fungal OTUs were optimized and annotated to the phylum classification level. For the composition of bacterial species, Actinobacteria, Chloroflexi, and Firmicutes were found to be the dominant phyla. Compared with CK, the relative abundance of Actinobacteria and Chloroflexi in T.L36 and T.B12 varieties under DT treatment increased significantly (Figure 5A). The relative abundance of Firmicutes decreased by 52.19% in T.L36, while that of T.B12 decreased by 95.51%, which is 43.32% more than that of T.L36. The results showed that Actinobacteria and Chloroflexi had better drought tolerance among the dominant bacteria. T.L36 drought-tolerant varieties had a strong preference for Firmicutes, while T.B12 drought-sensitive varieties preferred Actinobacteria (Figure 5A). For the composition of fungal species, Basidiomycota and Ascomycota were the dominant phyla. Compared with CK, the relative abundance of Ascomycota in T.L36 rhizosphere soil increased under DT treatment, while the relative abundance of Ascomycota in T.B12 decreased (Figure 5B). The relative abundance of Basidiomycota in T.B12 increased but decreased in T.L36. The relative abundances of Mortierellomycota and Glomeromycota in T.L36 rhizosphere soil under DT treatment increased by 266.13% and 620.17%, respectively, compared with CK, while the relative abundance of the above two phyla decreased in T.B12. Therefore, T.L36 drought-tolerant varieties have different preferences for Mortierellomycota, Glomeromycota, Ascomycota, and other fungi, while T.B12 drought-sensitive varieties prefer Basidiomycota (Figure 5B).

At the level of bacterial genus classification, the relative abundance of *Pedobacter* increased significantly in both T.L36 and T.B12. Compared with CK, the relative abundance of *Sphingomonas* under DT treatment increased by 2.83% and 168.08% in T.L36 and T.B12, respectively (Figure S7A). Under DT treatment, the abundance of *Fusarium* and *Pseudombrophila* in the rhizosphere soil fungi of T.L36 and T.B12 showed an increasing trend. Among them, *Fusarium* and *Pseudombrophila* increased by 69.64% and 2238.89% in T.L36 and 455.34% and 2.25% in T.B12, respectively. Under drought stress, *Pedobacter*, *Sphingomonas*, and *Fusarium* preferred T.B12 drought-sensitive varieties, while *Pseudombrophila* preferred T.L36 drought-tolerant varieties.

### 3.6. Analysis of Differential Species between Rhizosphere Microorganisms of Spring Wheat under Drought Stress

In this study, LEfSe analysis was used to further study the species with significant differences between T.L36 drought-resistant varieties and T.B12 drought-sensitive varieties of spring wheat rhizosphere soil samples under drought treatment (Figure S5B). In the bacterial community, there were four genera (*Bacillus*, *Spirosoma*, *Prevotellaceae*, *Chitinophaga*) with significant differences in T.L36 under CK treatment. There were four in T.B12 (*Faecalibaculum*, *Blautia*, *Alistipes*, *unidentified_Clostridiales*). Under DT treatment, there was one genus (*Gaiella*) with a significant difference in T.L36; there were five genera (*Pedobacter*, *Nocardioides*, *Streptomyces*, *Blastococcus*, *Solirubrobacter*) with significant differences in T.B12 (Figure S8A,B). In the fungal community, there were two genera (*Cutaneotrichosporon*, *Apiotrichum*) with significant differences in T.L36 under the CK treatment. There were five in T.B12 (*Coniothyrium*, *Didymella*, *Coniophora*, *Pleurotus*, *Valsa*). Five genera (*Pseudomrophila*, *Mortierella*, *unidentified*, *Acremonium*, *Chaetomium*) were significantly different in T.L36 under DT treatment. There was one (*unidentified*) in T.B12 (Figure S8C,D). The results showed that the types of T.L36 drought-resistant tolerant varieties and T.B12 drought-resistant sensitive varieties were different, and the enriched genera were also significantly different.

**Figure 6:**
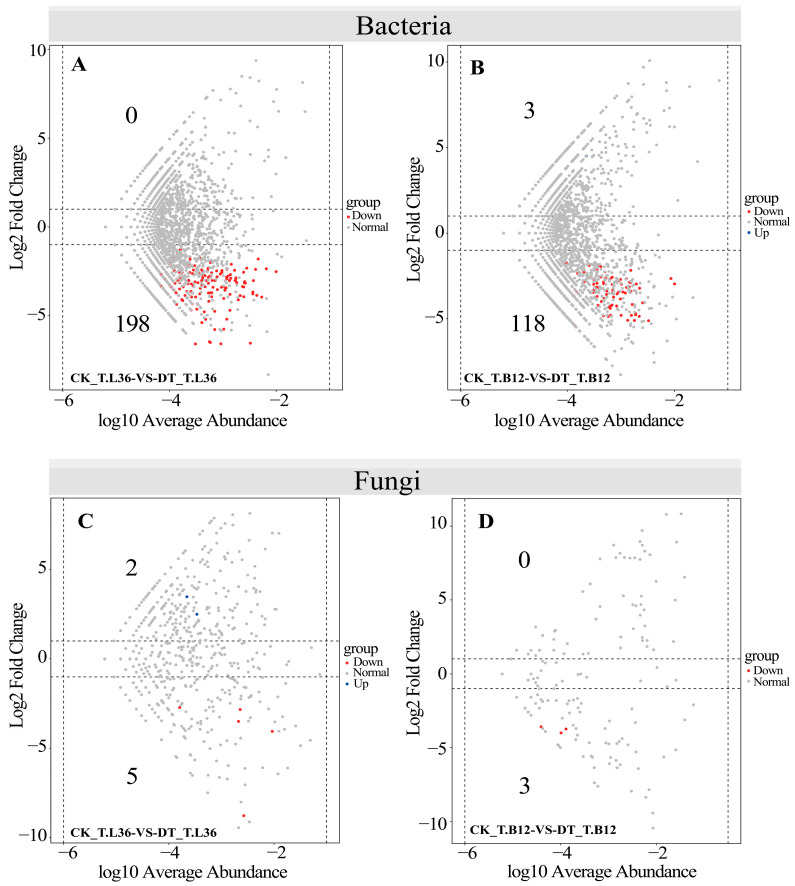
Compared with the control, bacterial and fungal OTUs were enriched and depleted in T.L36 and T.B12 spring wheat varieties under drought treatment. Each point represents an individual OTU, and the position along the y-axis represents a change in abundance multiples. The red dots represent depleted OTUs. The blue dots represent Enriched OTUs. The gray dots indicate OTUs with no significant difference. (**A**) CK_T.L36-VS-DT_T.L36 Bacterial Volcano Chart. (**B**) CK_T.B12-VS-DT_T.B12 Bacterial Volcano Chart. (**C**) CK_T.L36-VS-DT_T.L36 fungal volcano plot. (**D**) CK_T.B12-VS-DT_T.B12 Fungal Volcano Chart.

Furthermore, we also used the negative binomial distribution of OTU counts to analyze the differential abundance, with CK as the control and 0.01 as the critical value of the adjusted *p*-value (Figure 6). From CK to DT, the number of OTUs with decreased relative abundances detected in the bacterial community of T.L36 was 198 (Figure 6A and Table S3). Three OTUs with increased relative abundance detected in the bacterial community of T.B12 were OTU_634 (P_Firmicutes; g_*Brevibacillus*), OTU_926 (P_Firmicutes; f_Lachnospiraceae), and OTU_1828 (P_Bacteroidetes; f_Muribaculaceae); the number of OTUs with decreased relative abundance was 118 (Figure 6B and Table S3). In the fungal community, T.L36 had two OTUs with increased relative abundance, which were OTU_31 (p_Ascomycota; s_*Penicillium* sp.), OTU_65 (p_Ascomycota; s_*Sordariomycetes* sp.), and the number of OTUs with decreased relative abundance was five (Figure 6C and Table S4). The number of OTUs with decreased relative abundance in T.B12 was three, which were OTU_38 (p_Ascomycota; s_*Chaetomium_perlucidum*), OTU_116 (p_Ascomycota; s_*Periconia* sp.), OTU_377 (p_Ascomycota; s_*Leptosphaeria* sp.) (Figure 6D and Table S4). The above results further confirm that the effect of drought stress on the rhizosphere bacterial community composition was greater than that on the fungal community’s composition. *Brevibacillus* and *Lachnospiraceae* in Firmicutes were significantly enriched in T.B12 under drought stress. Ascomycota was significantly enriched in the rhizosphere fungi of T.L36 and T.B12, but the enriched genera and species were significantly different. Therefore, under drought stress, rhizosphere microorganisms prefer certain varieties and can resist adverse hazards by increasing or decreasing their relative abundance.

### 3.7. Co-Occurrence Network Analysis of Rhizosphere Microorganisms in Spring Wheat under Drought Stress

**Figure 7:**
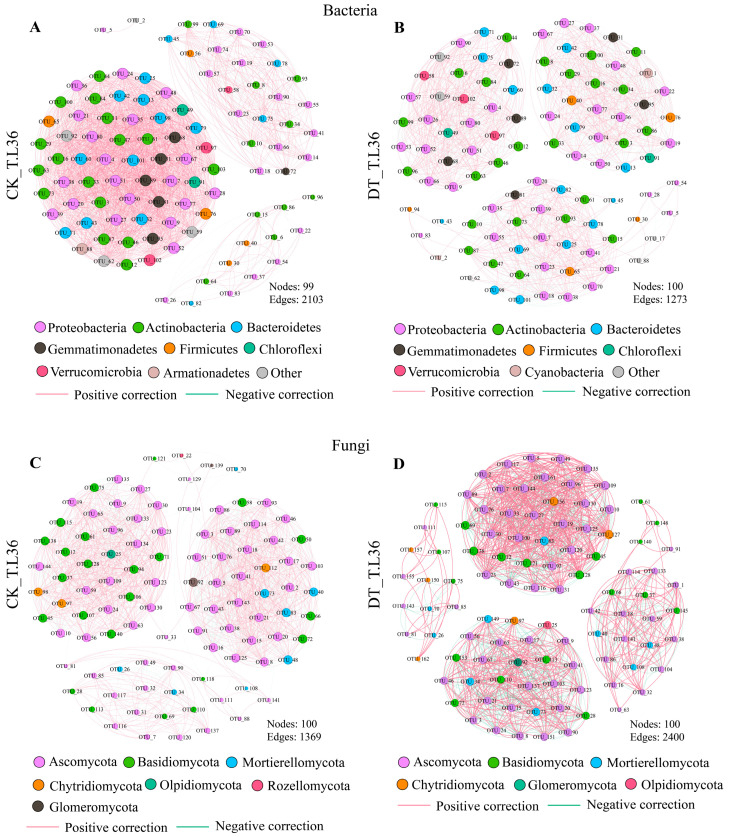
Rhizosphere microbial co-occurrence networks of T.L36 under drought stress. (**A**) CK_T.L36 rhizosphere bacteria co-occurrence network. (**B**) DT_T.L36 rhizosphere bacteria co-occurrence network. (**C**) CK_T.L36 rhizosphere fungi co-occurrence network. (**D**) DT_T.L36 rhizosphere fungi co-occurrence network. Connections indicate a significant correlation (Screening conditions: Spearman’s r > 0.9, *p* < 0.01). The pink line and the green line represent a positive correlation and negative correlation, respectively. Each node represents an OTU, the size of the node represents the degree, and the node is colored by the phylum.

We selected the top 100 OTUs for collinearity network analysis to explore the interaction between rhizosphere microorganisms of the T.L36 drought-tolerant variety (Figure 7) and T.B12 drought-sensitive variety (Figure S9) under drought stress. In general, the node numbers of bacteria and fungi in the two spring wheat varieties were similar, but the edge number and network density of bacteria were significantly higher than those of fungi, indicating that the network structure of bacteria was more complex than that of fungi. Visually, when compared with the control (CK), the bacterial network of T.L36 under drought (DT) treatment was loose, the number of edges decreased (Figure 7A,B), and the number of fungal edges increased (Figure 7C,D). The number of positive correlations between bacterial OTUs decreased from 1828 to 750; the negative correlation increased from 275 to 523; the number of positive correlations between fungal OTUs increased from 857 to 1398; the negative correlation increased from 512 to 1002, and the number of modular bacteria increased under DT treatment (CK-DT: 0.046–10.567), while fungi decreased (CK-DT: 1.544–0.621) (Table S5). The bacterial and fungal networks of T.B12 were loose, and the number of edges decreased. The number of positive correlations between bacterial OTUs decreased from 2420 to 664. The number of negative correlations increased from 180 to 634 (Figure S9A,B); the number of positive correlations between fungal OTUs decreased from 1354 to 1022. The number of negative correlations decreased from 866 to 232 (Figure S9C,D), the number of bacterial modules increased under DT treatment (CK-DT: 0.218–2.085) and the number of fungal modules (CK-DT: 1.377–1.764) also increased significantly (Table S5). The results show that drought reduced the correlation between the bacterial and fungal species of T.B12, and the network complexity was also reduced. The interaction between bacterial species was seriously restricted by drought stress. The rhizosphere bacterial community of the T.L36 drought-tolerant varieties have rich small-world properties, which ensures the stability of the microbial niche in adversity. Fungi can also resist drought damage by enhancing the positive correlation between species.

In the bacterial community, there were three dominant bacterial groups in T.L36 under DT treatment, which were OTU_65 (p_Firmicutes; g_*Bacillus*), OTU_102 (p_Verrucomicrobia; s_*bacterium_Ellin517*) and OTU_16 (p_Actinobacteria; g_*Blastococcus*) (Figure 7B). In the fungal community, there were three dominant bacterial groups in T.L36 under DT treatment, which were OTU_27 (p_Ascomycota; g_*Cladorrhinum*) and OTU_144 (p_Ascomycota; g_*Pseudogymnoascus*) (Figure 7D). In the bacterial community, the dominant bacterial groups of T.B12 under DT treatment included OTU_8 (p_Actinobacteria; g_*Glycomyces*) and OTU_63 (p_Actinobacteria; o_Solirubrobacterales) (Figure S9B). In the fungal community, the dominant bacterial groups of T.B12 under DT treatment included OTU_145 (p_Basidiomycota; s_*Tremellomycetes* sp.), OTU_45 (p_Basidiomycota; g_*Holtermanniella*) (Figure S9D). The above dominant bacteria and fungi showed close interactions with other microorganisms.

## 4. Discussion

### 4.1. Morphological and Physiological Changes in Spring Wheat under Drought Stress 

The drought resistance of plants is affected by many factors. When plants face drought stress, they can develop special physiological and metabolic mechanisms and produce special morphological structures to reduce water loss so that they can gradually adapt to or resist drought [61,62]. Among them, biomass and photosynthetic physiological parameters are the comprehensive response that plants have to drought stress and are also reliable indicators to evaluate the degree of drought stress and the drought resistance of plants [63,64,65]. The results of this study showed that the leaf wilting, drooping, and yellowing of spring wheat varieties was enhanced under drought (DT) treatment, and the plant height, fresh weight (FW), dry weight (DW), net photosynthetic rate (Pn), and stomatal conductance (Gs) of flag leaves were significantly decreased (*p* < 0.05). The results of our study are consistent with those of previous studies [33,66,67,68]. Under drought stress, the height of crop stems decreases, and the growth rate of leaves slows down. With drought stress, the inhibition of the crop leaf area, biomass, root length, and other indicators is more obvious, and the response of crop genotypes to drought stress shows individual differences [69,70,71,72]. The initial response of plants to drought stress is achieved by adjusting the stomatal aperture, which can maintain photosynthesis to a certain extent and prevent water loss in plants [61]. Our study found that the variation in the photosynthetic indices of drought-tolerant varieties (T.L36) under drought treatment was less than that of drought-sensitive varieties (T.B12). This result indicates that under certain drought conditions, drought-tolerant wheat genotypes show some protective mechanisms to maintain their growth needs as much as possible [73,74,75]. Furthermore, we also found that the POD and Pro contents of T.L36 varieties increased by 1.29% and 24.24%, respectively. Niu et al. [76] found that the POD activity of cotton increased under drought stress after the drought treatment of cotton. Singh et al. [77] suggested that there were differences in proline accumulation among different barley varieties (Hordeum vulgare) under the same leaf water potential, and it was positively correlated with field drought resistance. Slama et al. [78] found that water shortage leads to an increase in the proline content in traditional hard wheat varieties (Triticum durum Desf.), which is consistent with the results of this study.

The BADHb gene is edited to produce functional proteins in the process of plant stress resistance, which directly protects plant cells, and this gene is closely related to the drought resistance of wheat [79]. The Wdreb2 gene transmits signals and participates in the regulation of drought-related gene expression during plant stress resistance [80]. The results of this study show that TaWdreb2 and TaBADHb genes are highly expressed in T.D40, T.L36, and T.L33 and expressed at low levels in T.N2, T.B12, and T.F5 under drought treatment, which is consistent with the results of Egawa et al. [59] and Niazian et al. [60]. We hypothesized that under drought stress, spring wheat reduces stomatal conductance by closing or partially closing the stomata, regulating plant height, leaves, and other plant morphology, and reducing water evaporation; photoinhibition and photorespiration which produces a large amount of ROS accumulation, and causes damage to the cell membrane system of crops. Spring wheat with strong drought resistance actively upregulates the expression of TaWdreb2, TaBADHb, and other related genes, increases the activity of antioxidant enzymes such as POD, increases the content of water-soluble substances such as Pro in the cytoplasm, regulates the osmotic pressure inside and outside the cell, and maintains a relatively good water balance to maintain normal life activities.

### 4.2. Changes in Soil Nutrients and Microbiological Characteristics of Spring Wheat under Drought Stress

Carbon (C), nitrogen (N), and phosphorus (P) are essential elements for plant growth, energy storage and transport, stress resistance, and other biogeochemical material cycles [81]. Drought indirectly affects plant growth by changing soil water availability, nutrient content, soil microbial biomass, and enzyme activity [82,83,84]. Our study found that compared with CK, DT treatment significantly reduces the contents of TN, MBC, MBN, MBP, SOC, and S-ALP in six spring wheat fields, while the contents of TP, TK, and S-CAT in the soil were significantly increased (*p* < 0.05). Zhu et al. [85] also found that drought reduced the content of soil organic carbon and total nitrogen in the surface soil (0–10 cm) of alpine grassland compared with the control. Wang et al. [86] pointed out that reduced precipitation can significantly inhibit nutrient supply, microbial growth efficiency, and the decomposition of organic matter. The above studies indicate that drought usually has a negative impact on soil properties. However, some studies have found that drought can increase soil nutrient concentrations, soil organic carbon, total nitrogen, and available phosphorus, resulting in a decrease in soil nutrient mobility [84,87,88]. This is different from the results of this experimental study. A possible reason for this is that there are differences in crop types, site conditions, and treatment methods. In addition, soil microorganisms are the main driving force of soil nutrient cycling, which is affected by the type of interaction between crops and the soil environment. Different crops have different responses to drought, and there can be some differences in soil nutrient deficiency. Global environmental change factors such as drought and nutrient enrichment have been shown to reduce soil microbial biomass content [89]. Phosphatase is involved in the transformation of organic and inorganic phosphorus compounds in the soil [90], and its activity is an important factor in maintaining and controlling the rate of the phosphorus cycle in soil. Dodor et al. [91] and Sardans et al. [92] noted that a decrease in alkaline phosphatase activity under drought stress indicated that a decrease in phosphorus availability harmed water use efficiency and indirectly aggravated the drought effect. In short, under drought stress, spring wheat may affect soil indicators through the microenvironment. The presence of these microenvironments has more water-soluble compounds that are conducive to maintaining water in the soil, thereby sustaining the activity of soil microorganisms to a certain extent. Spring wheat with strong drought resistance has more advantages than drought-sensitive spring wheat in this respect. Therefore, soil nutrients, soil enzyme activity, and soil microbial biomass content can maintain a relatively good balance to ensure the basic needs of plant growth.

### 4.3. Response of Rhizosphere Microorganisms of Drought-Tolerant Spring Wheat to Drought Stress

The rhizosphere is an area where there is a strong interaction between crop roots, soil, and microorganisms [93]. Studies have shown that drought has a significant impact on the composition and activity of the plant rhizosphere microbiome, and the composition of rhizosphere microorganisms is regulated by host genotypes [27,94,95]. Microorganisms interact in a variety of ways, including symbiosis, competition, and cooperation mediated by signaling molecules or metabolites, to assist crops in playing a key role [96]. Ureases secreted by microorganisms can also participate in urea hydrolysis and improve the soil microenvironment [97]. Moreover, rhizosphere microorganisms can also promote the growth and development of host crops by improving root structure and water and nutrient uptake [93]. This study found that there was no significant difference in the Shannon index between the same variety of spring wheat under control (CK) and drought (DT) treatments (*p* > 0.05), but the bacterial Shannon index of T.L36 increased, which was the largest increase, while the fungal Shannon index of T.B12 decreased by 5.57%. The results show that the variation in the Shannon index was related to the variety and treatment, and the rhizosphere microorganisms had a certain degree of drought resistance. Liu et al. [98] also found that there was no significant difference in microbial alpha diversity after the drought treatment of sugarcane. This is similar to our results, indicating that crop rhizosphere microorganisms can resist a certain degree of drought interference, but this anti-interference ability and the degree of stress or the length of time remain to be further explored.

Different environments induce different changes in microbial communities, and changes in microbial communities can further affect the biogeochemical reactions of various substances [99]. In this study, we found that Actinobacteria, Chloroflexi, and Firmicutes were the dominant bacterial phyla under drought treatment. The abundance of Actinobacteria and Chloroflexi increased significantly after stress between resistant and susceptible varieties, indicating that these bacteria had a level of drought resistance. Actinobacteria, Chloroflexi, and Firmicutes are Gram-positive bacteria with a thick cell wall and the ability to form spores and resist certain stresses [100,101], which can be the reason why they can become dominant groups in arid environments. In addition, Actinobacteria are mostly saprophytic bacteria that grow in drought and other stressful environments, are related to the formation of soil structure [102,103], and can contribute to crop growth and development. The relative abundance of Firmicutes decreased significantly under drought stress, but there were significant differences among varieties, and T.B12 decreased by 95.51%. It was suggested that although Firmicutes can resist dehydration and extreme environments and easily survive in dry soil [104], their ability to resist drought may be limited and also related to host crop genotypes, while their interaction with preferred crops may maximize drought resistance. Actinobacteria were significantly enriched in relatively dry environments or under drought conditions [27,105]. Therefore, Firmicutes and Actinobacteria may have the ability to assist crops in drought resistance. 

Similarly, fungi also play an important role in the stress-resistant growth of crops [106]. Basidiomycota and Ascomycota are the most abundant fungal phyla in plant rhizosphere soil [107,108,109], and Khan et al. [106] found that Ascomycota and Basidiomycota were significantly enriched in the rhizosphere of plants in arid environments, which is consistent with the results of this study. *Fusarium* is a well-known pathogen that causes destructive root rot in plants, causing plants to wilt and seriously damage plant and crop health [110,111,112,113]. *Sphingomonas* can promote plant growth and alleviate abiotic stress [114,115,116]. *Pedobacter* can synthesize indole derivatives, benzoic acid derivatives, and thiobacillin, which can regulate wheat growth and resist wheat stripe rust [117]. Different strains have different roles and functions, and under drought stress, there are obvious differences in the enrichment of strains of different resistant spring wheat varieties caused by differences in varieties. For drought-resistant sensitive varieties, especially susceptible varieties of pathogenic bacteria, in the actual production process, attention should be given not only to drought hazards but also to preventing the harm caused by pathogenic bacteria. 

In this study, species differences at the genus level and OTU level were analyzed via LEfSe analysis and OTU count negative binomial distribution. There were five significantly different species of T.L36, which were *Bacillus*, *Spirosoma*, *Prevotellacea*, *Chitinophaga*, and *Gaiella*. There were also five species with significant differences in T.B12 under drought treatment, including *Pedobacter*, *Nocardioides*, *Streptomyces*, *Blastococcus*, and *Solirubrobacter*. Two OTUs with an increased relative abundance of T.L36 in the fungal community were OTU_31 (p_Ascomycota; s_*Penicillium* sp.) and OTU_65 (p_Ascomycota; s_*Sordariomycetes* sp.), while T.B12 had three OTUs with decreased relative abundance, which were OTU_38 (p_Ascomycota; s_*Chaetomium_perlucidum*), OTU_116 (p_Ascomycota; s_*Periconia* sp.), OTU_377 (p_Ascomycota; s_*Leptosphaeria* sp.). It has been reported that beneficial bacteria such as *Bacillus*, *Pseudomonas*, *Streptomyces*, *Nocardioides*, and *Chitinophaga* exhibit plant growth-promoting (PGP) traits in different crops under drought stress [118,119] and drought-tolerant PGPR can promote plant growth and improve drought stress [120]. Different *Penicillium* species provide a variety of nutrients and plant hormones and actively interact with plant roots. Khan et al. [121] showed that GA production by *Penicillium* reddened LK6 under drought stress and could promote the normal growth and development of pepper (*Capsicum annuum*). It has also been reported that *Simplicillium* sp. can be used as a biocontrol agent for plants [122]. Therefore, under drought stress, both drought-tolerant varieties (T.L36) and drought-sensitive varieties (T.B12) actively recruit beneficial microorganisms and maintain their growth through the life activities of such beneficial microorganisms to help resist drought hazards. This could be a survival strategy that has evolved in plants in a complex growth environment for a long time. In addition, there is an interesting phenomenon that the relative abundance of OTU_38, OTU_116, and OTU_377 pathogens in T.B12 drought-sensitive varieties decreased under drought stress, while the analysis of species composition found that the relative abundance of *Fusarium* increased by 455.34% under drought treatment, and there could be antagonistic constraints between species. Whether this is related to plant evolutionary survival strategies needs further study. The network structure of bacteria was more complex than that of fungi. The complexity of the T.B12 network decreased under drought stress, and the interaction between bacterial species was seriously restricted by drought. The rhizosphere bacterial community of T.L36 drought-tolerant varieties has rich small-world properties, which ensures the stability of the microbial niche in adversity.

**Figure 8:**
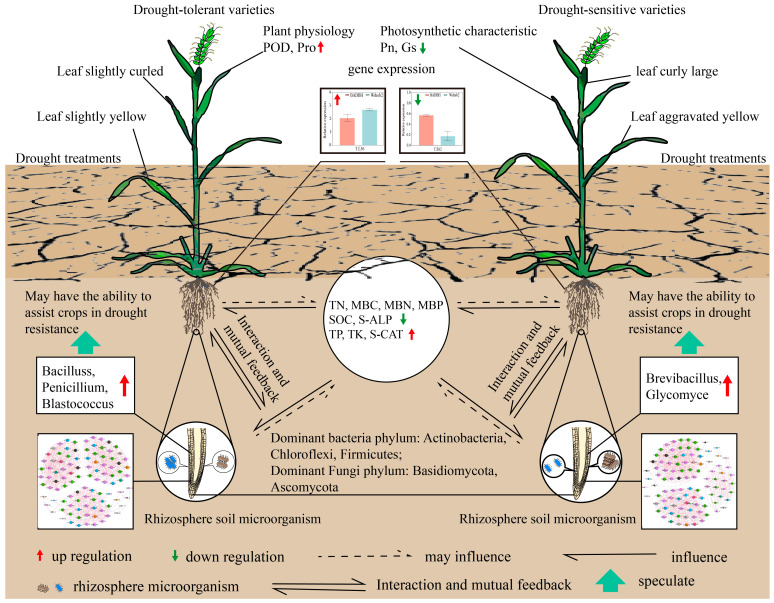
Summary diagram of the response mechanism of spring wheat rhizosphere microorganisms to drought stress.

In summary, we drew a summary map of the response of spring wheat rhizosphere microorganisms to drought stress based on the research results. Under drought stress, spring wheat actively regulated plant morphology and photosynthetic characteristics such as plant height and stomatal conductance upregulated the expression of drought-related genes in the body, increased the activity of antioxidant enzymes such as POD, increased the content of water-soluble substances such as Pro in the cytoplasm, resisted ROS accumulation hazards, regulated intracellular and extracellular osmotic pressure, and avoided water imbalance in the body to maintain physiological metabolic needs. In addition, drought-tolerant spring wheat can increase the relative abundance of rhizosphere drought-related beneficial microorganisms such as Actinobacteria and Firmicutes, regulate soil nutrients and microbiological properties, and improve the soil microenvironment, while plant–soil microorganisms cooperate to resist drought hazards (Figure 8).

## 5. Conclusions

The results show that compared with the control (CK), there were significant differences in plant morphology, physiological parameters, gene expression, soil nutrients, and soil microbiological traits in the six spring wheat varieties under drought (DT) treatment (*p* < 0.05). Drought-tolerant varieties can actively upregulate the expression of drought-related genes such as *TaWdreb2* and *TaBADHb*, increase the activity of antioxidant enzymes such as peroxidase (POD) and increase the content of water-soluble substances such as proline (Pro) in the cytoplasm; reduce the concentration of intracellular reactive oxygen species; regulate the osmotic pressure inside and outside the cell; maintain the balance of soil nutrients, soil enzyme activity, and other indicators in a better state; and enhance their adaptability to drought. The network structure of bacteria is more complex than that of fungi. The complexity of the drought-sensitive varieties T.B12 network decreased under drought stress, and the interaction between bacterial species was severely restricted via drought. The rhizosphere bacterial community of the drought-tolerant variety T.L36 has rich small-world network properties, which ensures the stability of the microbial niche under adversity. Under drought treatment, the T.L36 and T.B12 varieties were enriched with beneficial microorganisms such as Actinobacteria and Chloroflexi, while OTU_65 (p_Firmicutes; g_*Bacillus*), OTU_31 (p_Ascomycota; s_*Penicillium* sp.), OTU_16 (p_Actinobacteria; g_*Blastococcus*); T.B12 OTU_634 (P_Firmicutes; g_*Brevibacillus*), OTU_8 (p_Actinobacteria; g_*Glycomyce*) and other bacteria were dominantly enriched and played a key role in improving the drought resistance of spring wheat. In general, our results support the enrichment of differential beneficial microorganisms in drought-tolerant spring wheat varieties (T.L36) and drought-sensitive varieties (T.B12). These beneficial microorganisms may have the ability to assist crops in resisting drought damage.

## Figures and Tables

**Table 1:** Bacterial and fungal diversity in spring wheat rhizosphere soil under drought treatment.

Category	Sample Name	Shannon Index	|CK-DT|
Bacteria	CK_T.L36	7.722 ± 1.738 a	1.835
	DT_T.L36	9.557 ± 0.137 a	
	CK_T.B12	7.619 ± 1.637 a	1.742
	DT_T.B12	9.361 ± 0.157 a	
Fungi	CK_T.L36	5.892 ± 1.138 a	0.180
	DT_T.L36	6.072 ± 0.100 a	
	CK_T.B12	4.865 ± 0.472 a	0.271
	DT_T.B12	4.594 ± 1.014 a	

Note: The lowercase letters on the table indicate whether the Shannon index for rhizosphere microorganisms of the same spring wheat under different treatments was significant at the *p* < 0.05 level.

## Data Availability

The fungal and bacterial raw DNA sequences used in this study were deposited in the Sequence Read Achieve (SRA) of the NCBI database under the accession number PRJNA995401 for open access.

## Supplementary Materials

The following supporting information can be downloaded at: https://www.mdpi.com/article/10.3390/plants12203650/s1, Figure S1: The distribution map of daily precipitation and daily average temperature during the whole growth period of spring wheat in 2019; Figure S2: Monitoring of soil mass water content under drought treatment; Figure S3: Effects of drought stress on fresh weight, and dry weight of spring wheat; Figure S4: Effects of drought stress on soil organic carbon and enzyme activity of spring wheat; Figure S5: Effects of drought stress on rhizosphere microbial–rarefaction curves of T.L36 and T.B12; Figure S6: PCOA analysis of T.L36 and T.B12 rhizosphere microbial community composition under drought stress; Figure S7: T.L36 and T.B12 under drought stress Species composition at the genus level; Figure S8: Lefse analysis of species with significant differences between T.L36 and T.B12 rhizosphere microbial groups under drought stress; Figure S9: Rhizosphere microbial co-occurrence networks of T.B12 under drought stress; Table S1: Spring wheat variety information; Table S2: Primer sequence information; Table S3: Compared with the control, the specific data table of bacterial OTUs enrichment and depletion in T.L36 and T.B12 spring wheat varieties under drought treatment; Table S4: Compared with the control, the specific data table of fungal OTUs enrichment and depletion in T.L36 and T.B12 spring wheat varieties under drought treatment; Table S5: The topological characteristics of the co-occurrence network of rhizosphere microbial communities of different spring wheat varieties under drought stress (Figure 7 and Figure S9).
