# Supplementary material for: Drought Sensitivity of Spring Wheat Cultivars Shapes Rhizosphere Microbial Community Patterns in Response to Drought
Source: Plants (Basel). 2023 Oct 23;12(20):3650. doi: 10.3390/plants12203650 (PMC10609721; doi:10.3390/plants12203650)
Supplement: Supplementary file 1 [file plants-12-03650-s001.zip › plants-2578722-supplementary.pdf]

Table S1. Spring wheat variety information

Variety	Abbreviation	Approval year	Breeding organization
Nongmai No. 2	T.N2	National authorized wheat Variety 2006030	Crop Research Institute of Inner Mongolia Academy of Agricultural Sciences
Bafeng No. 5	T.N5	National authorized wheat Variety 2009028	Bayannaoer Academy of Agricultural and Animal Husbandry Sciences
Bamai No. 12	T.N12	Inner Mongolia authorized wheat variety 2015002	Bayannaoer Academy of Agricultural and Animal Husbandry Sciences
Longmai No. 33	T.N33	National authorized wheat Variety 2010022	Crop Breeding Institute of Heilongjiang Academy of Agricultural Sciences
Longmai No. 36	T.N36	Heilongjiang authorized wheat Variety 2013001	Crop Breeding Institute of Heilongjiang Academy of Agricultural Sciences
Dingxi No. 40	T.N40	National authorized wheat Variety 2009032	Dingxi Dry Farming Research and Extension Center, Gansu Province

Table S2. Primer sequence information

Primer name	Primer sequence (5'- 3')	Product size (bp)	Annealing temperature (℃)
Wdreb2	F: AGATGTTGCTTCTTCCTTGCC<br>R: GATGTGCTCCTTGAAATGCTTG	162	60
BADHb	F: GTGGACTCTATTTGGGTGCTTTTGG<br>R: GCACCTTCACTTTTAGCATTCGCTAC	231	60
Actin	F: CTTGTATGCCAGCGGTCGAACA<br>R: CTCATAATCAAGGGCCACGTA	241	60

Table S3. Compared with the control, the specific data table of bacterial OTUs enrichment and depletion in T.L36 and T.B12 spring wheat varieties under drought treatment.

Category	Sample comparison	OTU_number	Taxa	log2FoldChange	p_value	Log10 Average Abundance	group	number
Bacteria	CK_T.B12-vs-DT_T.B12	OTU_634	P_Firmicutes; c_Bacilli; o_Bacillales; f_Paenibacillaceae; g_Brevibacillus; s_Brevibacillus_reuszeri	3	0.007762603	-4.545257621	Up	3
		OTU_926	P_Firmicutes; c_Clostridia; o_Clostridiales; f_Lachnospiraceae	4.502500341	0.000856072	-3.648241782	Up	
		OTU_1828	P_Bacteroidetes; c_Bacteroidia; o_Bacteroidales; f_Muribaculaceae	3.807354922	0.001498449	-4.022378876	Up	
	CK_T.L36-vs-DT_T.L36	A total of 198 Depieted OTUs were identified						
	CK_T.B12-vs-DT_T.B12	A total of 118 Depieted OTUs were identified						

Table S4. Compared with the control, the specific data table of fungal OTUs enrichment and depletion in T.L36 and T.B12 spring wheat varieties under drought treatment.

Category	Sample comparison	OTU_number	Taxa	log2FoldChange	p_value	Log10 Average Abundance	group	number
Fungi	CK_T.L36-vs-DT_T.L36	OTU_31	p__Ascomycota;c__Eurotiomycetes;o__Eurotiales;f__Aspergillaceae;g__Penicillium;s__Penicillium_sp	3.48112669	0.003276175	-3.655451578	Up	2
		OTU_65	p__Ascomycota;c__Sordariomycetes;o__unidentified_Sordariomycetes_sp;f__unidentified_Sordariomycetes_sp;g__unidentified_Sordariomycetes_sp;s__Sordariomycetes_sp	2.497499659	0.007990344	-3.465695995	Up	
	CK_T.B12-vs-DT_T.B12	OTU_38	p__Ascomycota;c__Sordariomycetes;o__Sordariales;f__Chaetomiaceae;g__Chaetomium;s__Chaetomium_perlucidum	-4	0.003496578	-3.987295521	Down	3
		OTU_116	p__Ascomycota;c__Dothideomycetes;o__Pleosporales;f__Periconiaceae;g__Periconia;s__Periconia_sp	-3.736965594	0.00159644	-3.885305983	Down	
		OTU_377	p__Ascomycota;c__Dothideomycetes;o__Pleosporales;f__Leptosphaeriaceae;g__Leptosphaeria;s__Leptosphaeria_sp	-3.584962501	0.009963864	-4.404831086	Down	
	CK_T.L36-vs-DT_T.L36	A total of 5 Depieted OTUs were identified						
	CK_T.B12-vs-DT_T.B12	A total of 3 Depieted OTUs were identified						

Table S5. The topological characteristics of the co-occurrence network of rhizosphere microbial communities of different spring wheat varieties under drought stress (Figure 7 and S9).

**category**	**Bacteria**				**Fungi**			
Sample	CK_T.L36	DT_T.L36	CK_T.B12	DT_T.B12	CK_T.L36	DT_T.L36	CK_T.B12	DT_T.B12
Nodes	99	100	98	98	100	100	100	99
Edges	2103	1273	2600	1298	1369	2400	2220	1254
Average degree	53.061	26.49	42.485	25.46	22.2	25.333	27.38	24
Average weighted degree	45.714	1.224	125.495	18.16	9.76	63.838	27.6	7.92
Network diameter	1	1	1	1	1	1	1	1
Graph density	0.547	0.273	0.434	0.257	0.224	0.259	0.277	0.242
Modularity	0.046	10.567	0.218	2.085	1.544	0.621	1.377	1.764
Average clustering coefficient	1	1	1	1	1	1	1	1
average path length	1	1	1	1	1	1	1	1

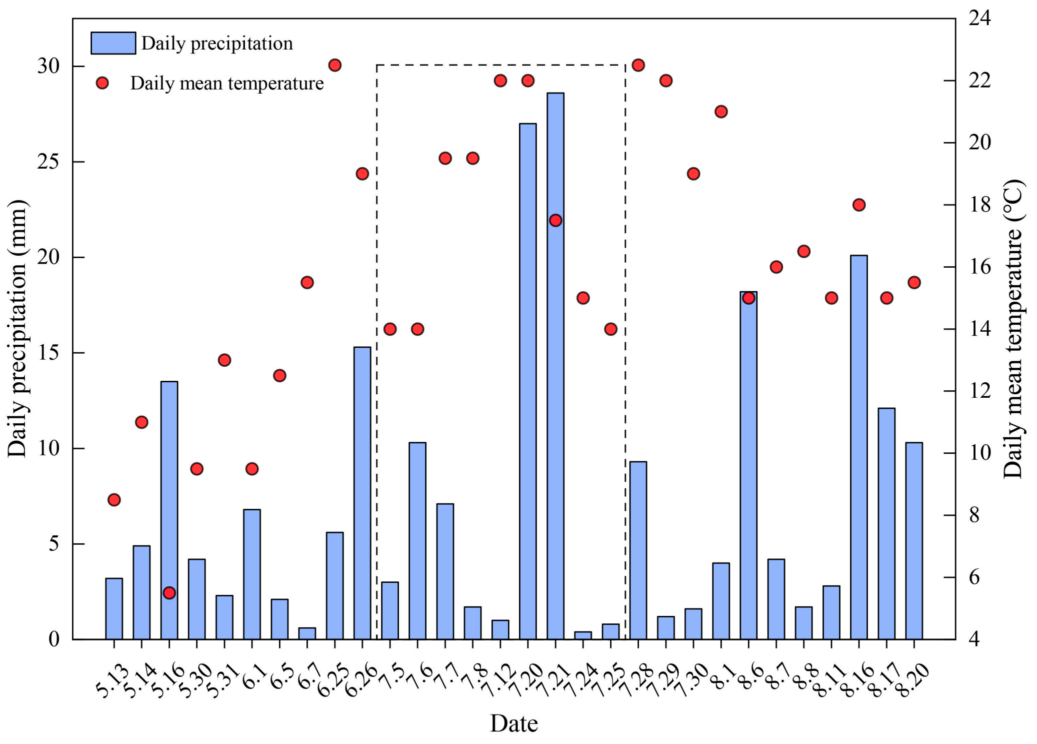

Figure S1. The distribution map of daily precipitation and daily average temperature during the whole growth period of spring wheat in 2019

Note: The dotted line frame in the picture is that the water control treatment time for the dry shed rain control - regulate water replenishment is from July 5 to July 25.

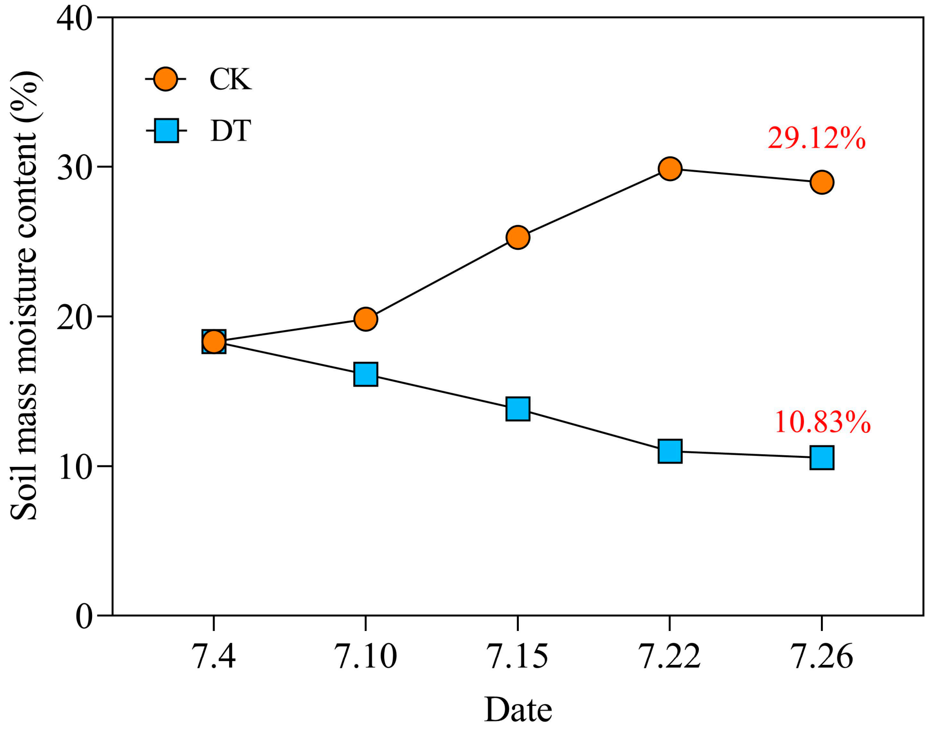

Figure S2. Monitoring of soil mass water content under drought treatment

Note: The control treatment (CK) was water replenishing twice (July 11, and July 16), supplementary water amount: each treatment was repeated 3 times, totaling 2 m^3.

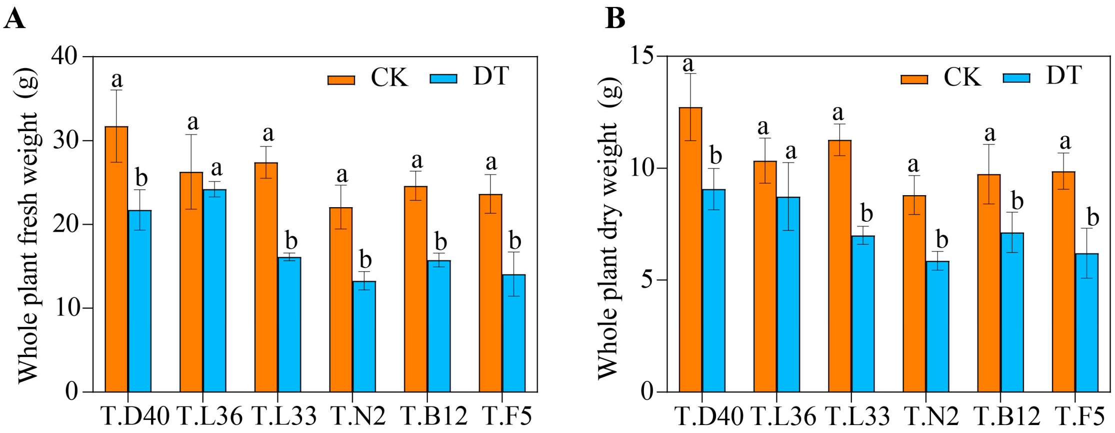

Figure S3. Effects of drought stress on fresh weight, and dry weight of spring wheat

Note: (**A**) Whole plant fresh weight (FW). (**B**) Whole plant dry weight (DW). Different letters indicate significant differences at the 0.05 level

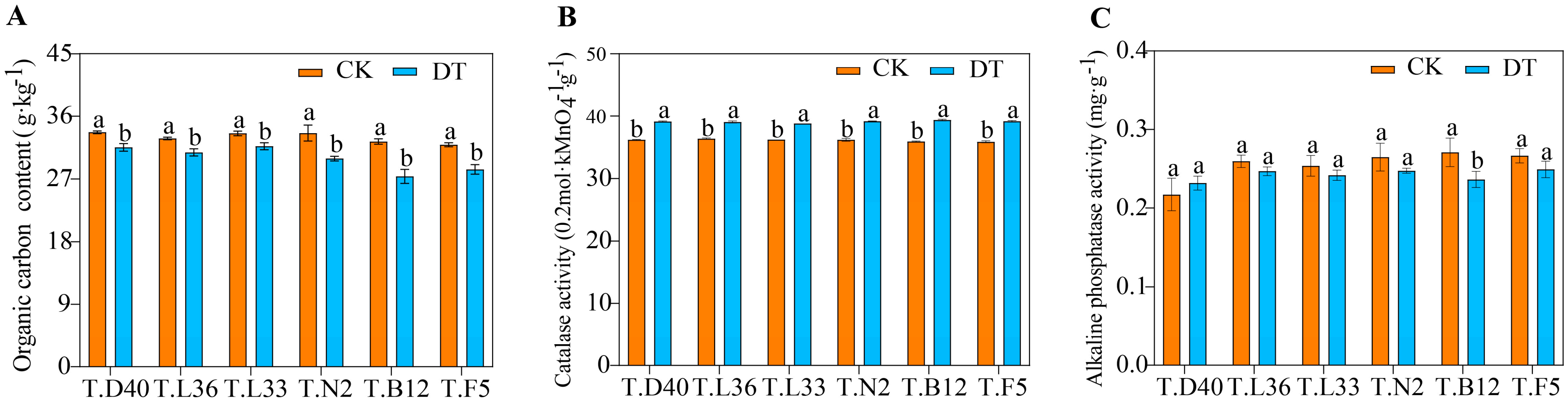

Figure S4. Effects of drought stress on soil organic carbon and enzyme activity of spring wheat

Note: (**A**) Organic carbon content (SOC). (**B**) Catalase activity (S-CAT). (**C**) Alkaline phosphatase activity (S-ALP). Different letters indicate significant differences at the 0.05 level.

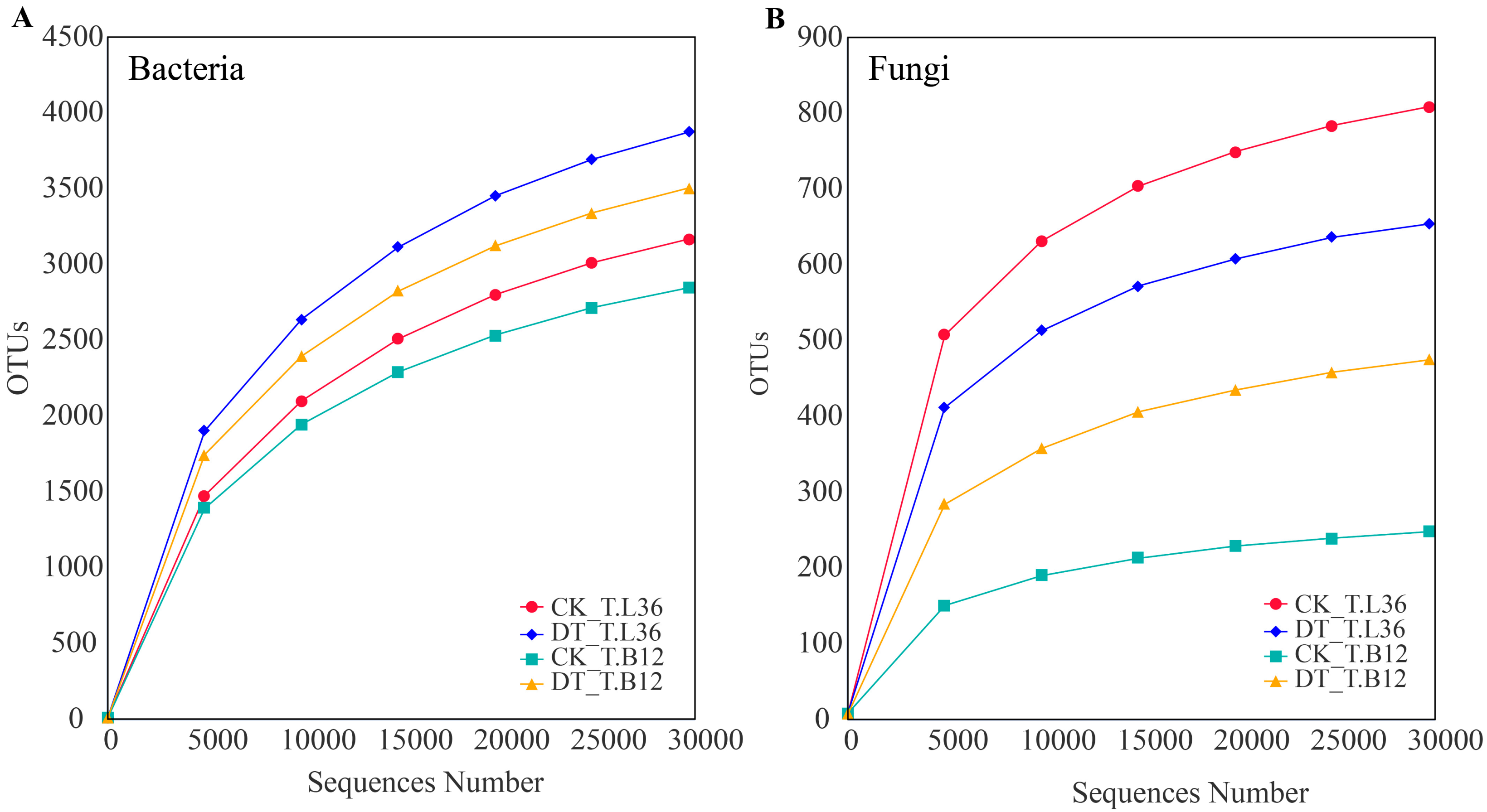

Figure S5. Effects of drought stress on rhizosphere microbial rarefaction curves of T.L36 and T.B12

Note: (**A**) Rhizosphere bacterial rarefaction curve. (**B**) Rhizosphere fungi rarefaction curve.

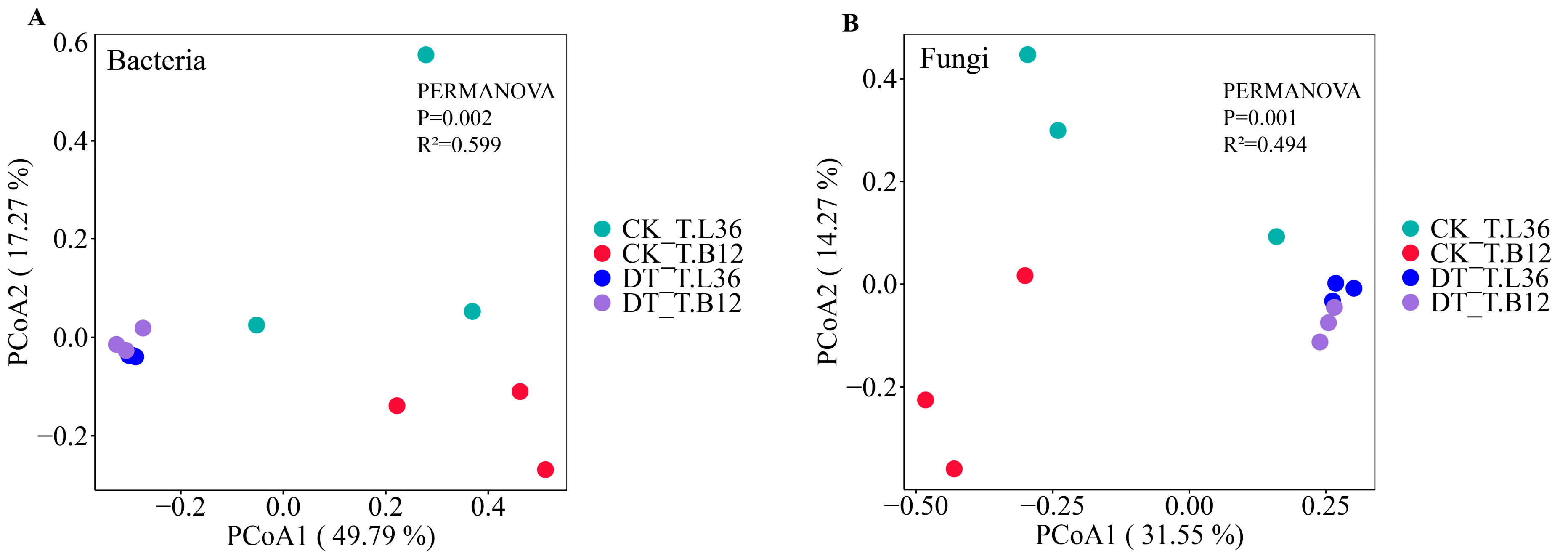

Figure S6. PCOA analysis of T.L36 and T.B12 rhizosphere microbial community composition under drought stress

Note: (**A**) Rhizosphere bacterial PCOA analysis. (**B**) Rhizosphere fungi PCOA analysis.

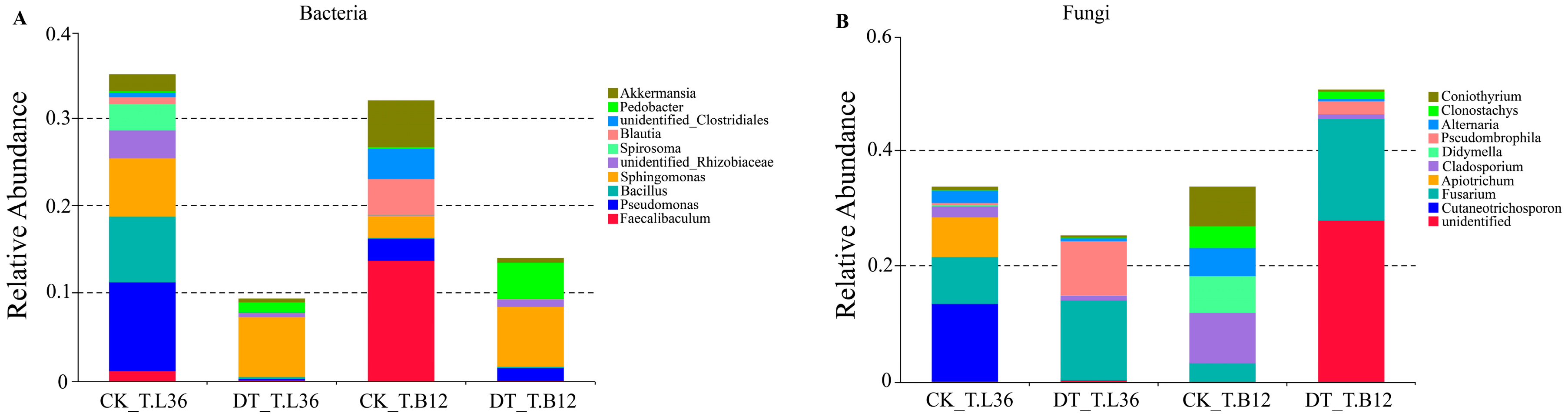

Figure S7. T. L36 and T. B12 under drought stress Species composition at the genus level

Note: (**A**) Species composition of rhizosphere bacteria at the genus level. (**B**) Species composition rhizosphere fungi at the genus level.

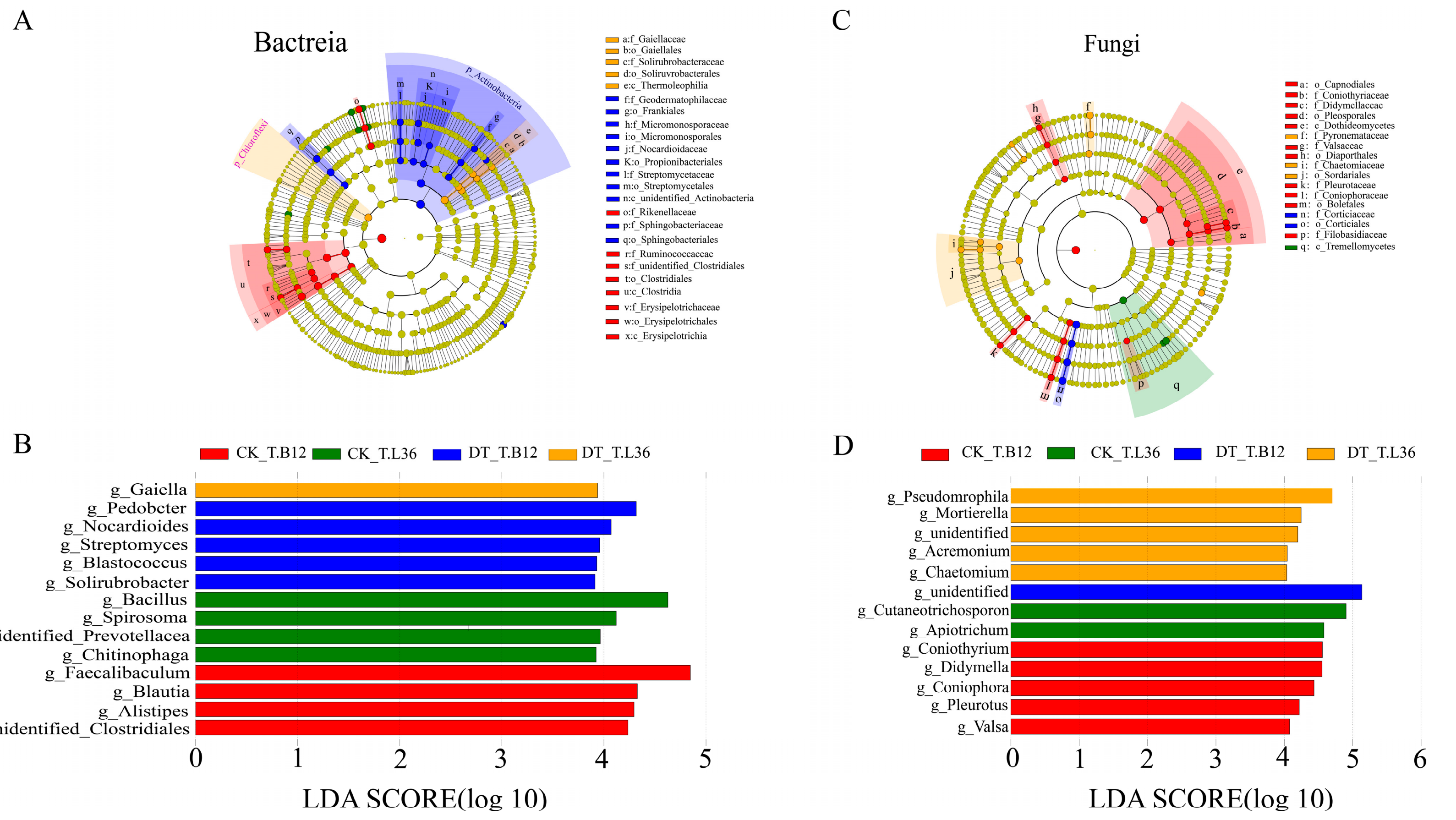

Figure S8. Lefse analysis of species with significant differences between T.L36 and T.B12 rhizosphere microbial groups under drought stress

Note: (**A**) Bacterial Lefse analysis evolutionary branch diagram. (**B**) Columnar diagram of LDA analysis of bacteria, LDA score of 3.9, *P<0.05*. (**C**) Fungal Lefse analysis evolutionary branch diagram. (**D**) Columnar diagram of LDA analysis of fungi, LDA score of 3.9, *P<0.05*.

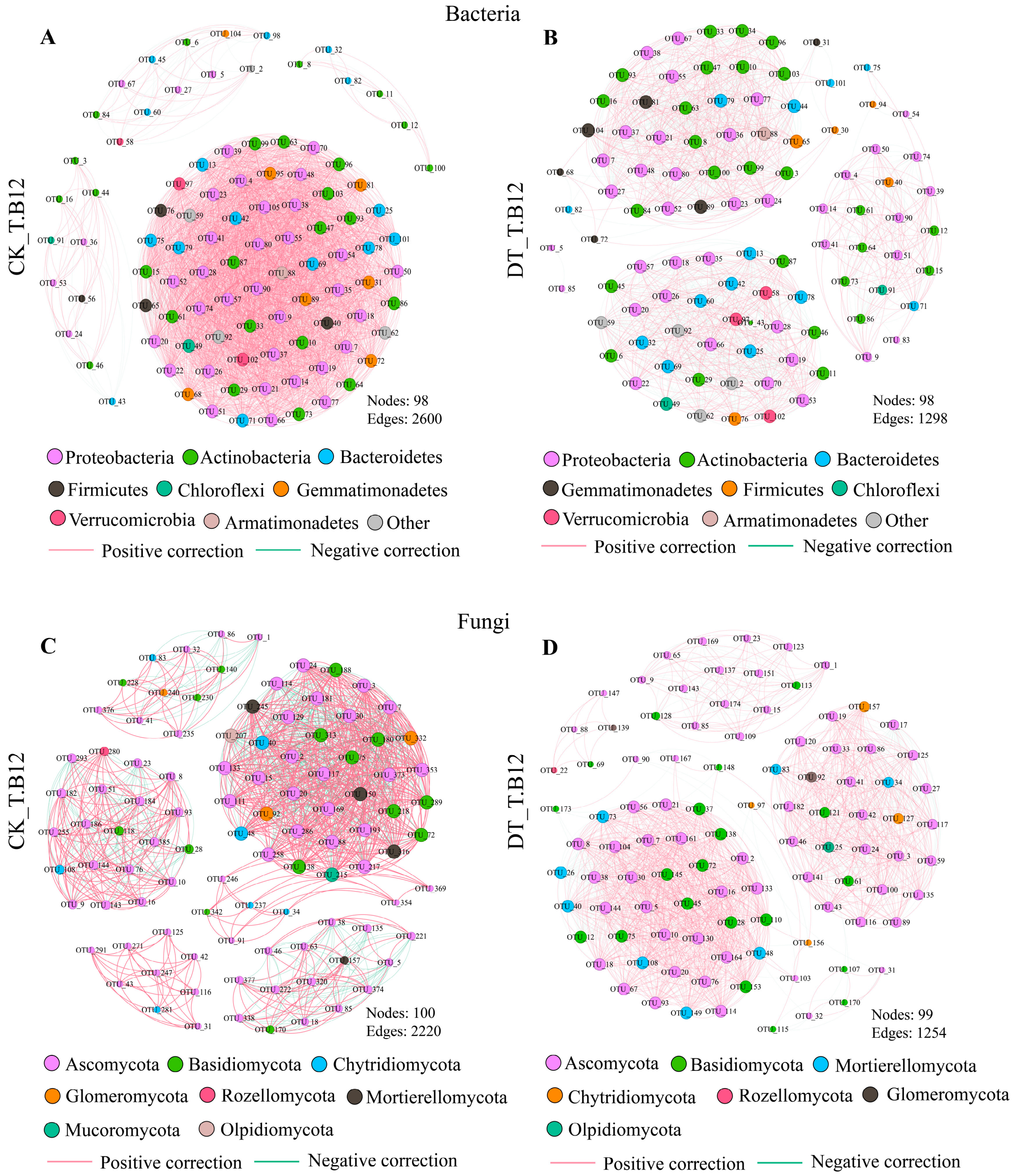

Figure S9. Rhizosphere microbial co-occurrence networks of T.B12 under drought stress.

Note: (**A**) CK _ T.L36 rhizosphere bacteria co-occurrence network. (**B**) DT _ T.L36 rhizosphere bacteria co-occurrence network. (**C**) CK _ T.L36 rhizosphere fungi co-occurrence network. (**D**) DT _ T.L36 rhizosphere fungi co-occurrence network. Connections indicate significant correlation (Screening conditions: Spearman's r > 0.9, $P < 0.01$); The pink line and the green line represent positive cor-relation and negative correlation, respectively. Each node represents an OTU, the size of the node represents the degree, and the node is colored by the phylum.
